# Hepcidin and Erythroferrone Correlate with Hepatic Iron Transporters in Rats Supplemented with Multispecies Probiotics

**DOI:** 10.3390/molecules25071674

**Published:** 2020-04-05

**Authors:** Katarzyna Skrypnik, Paweł Bogdański, Magdalena Sobieska, Joanna Suliburska

**Affiliations:** 1 Institute of Human Nutrition and Dietetics, Poznań University of Life Sciences, Wojska Polskiego St. 31, 60-624 Poznań, Poland; jsulibur@up.poznan.pl; 2 Department of Treatment of Obesity, Metabolic Disorders and Clinical Dietetics, Poznań University of Medical Sciences, Szamarzewskiego St. 84, 60-569 Poznań, Poland; pawelbogdanski73@gmail.com; 3 Department of Physiotherapy, Chair for Physiotherapy and Rehabilitation, Poznań University of Medical Sciences, 28. Czerwca 1956r St. 135/147, 61-545 Poznań, Poland; msobieska@ump.edu.pl

**Keywords:** probiotic supplementation, iron, hepcidin, erythroferrone, divalent metal transporter 1, transferrin receptor

## Abstract

The influence of probiotic supplementation on iron metabolism remains poorly investigated. However, a range of studies, especially on *Lactobacillus plantarum* 299v (Lp229v), have indicated a possible positive impact of probiotics on iron absorption. The aim of the study was to determine the effect of multistrain probiotic supply on iron balance. Thirty Wistar rats were randomized into three groups: placebo (KK group), and multistrain probiotic per os in a daily dose of 2.5 × 10^9^ colony forming units (CFU) (PA group) or 1 × 10^10^ CFU (PB group). Multistrain probiotic consisted of nine bacterial strains: *Bifidobacterium bifidum* W23, *B. lactis* W51, *B. lactis* W52, *Lactobacillus acidophilus* W37, *L. brevis* W63, *L. casei* W56, *L. salivarius* W24, *Lactococcus lactis* W19, and *Lc. lactis* W58, in equal proportions. After six weeks, blood and organ samples were collected. No differences were found between the three groups in terms of serum concentrations of hepcidin (HEPC), lactoferrin (LTF), homocysteine (HCY), ferritin (Ft), or erythroferrone (ErFe), or in liver content of divalent metal transporter 1 (DMT1), transferrin receptors 1 and 2 (TfR), or ZRT/IRT-like protein 14 (ZIP14) proteins. In the overall sample, positive correlations were noted between the serum concentrations of hepcidin and lactoferrin, and hepcidin and ferritin; serum concentration of hepcidin and DMT1 and TfR1 in the liver; and serum concentration of erythroferrone and TfR2 in the liver. The correlations of serum hepcidin and erythroferrone with liver DMT1 and TfR represent significant mechanisms of Fe homeostasis. Our study has shown that multistrain probiotic supplementation used in the experiment did not disrupt the biochemical and hepatic regulatory processes of Fe balance and did not demonstrate significant influence on selected parameters of Fe metabolism.

## 1. Introduction

Correct iron (Fe) metabolism enabling the proper functioning of the body is crucial for a range of physiological processes. As a component of hemoglobin and myoglobin particles, Fe takes part in oxygen and energy balance [1]. Fe plays an essential role in catalytic processes involving enzymes such as peroxidase, cytochromes, and catalase [1]. Maintenance of a healthy state requires sufficient stores of this microelement, yet Fe excess is a factor leading to such detrimental states as intensified oxidative stress [2], inflammation [3], liver [4] and cardiac [5] dysfunction, kidney injury [6], neurodegeneration [2], adipose tissue dysfunction [7], glucose metabolism disorders [8], and disrupted gene expression [9]. On the other hand, Fe deficiency leads to anemia [10].

The human body contains about 3–5 g of Fe [11]. Intestinal Fe absorption takes place mainly in the duodenum and upper jejunum. Inorganic dietary nonheme ferric iron is reduced by membrane-bound ferrireductase (DCYTB) and then absorbed by duodenal enterocytes via divalent metal transporter 1 (DMT1) [12]. However, our understanding of this reduction process is not complete, and the role of DCYTB is not clear [13]. Fe is released from the enterocytes to the bloodstream by basolateral exporter ferroportin. Fe is transported in the blood in the form of transferrin [14]. Transferrin binds to transferrin receptor 1 (TfR1), which is especially highly expressed on erythroblast surfaces, and internalizes into cells [15]. It is hypothesized that simultaneously, TfR1-associated protein (HFE) is translocated from TfR1 into transferrin receptor 2 (TfR2), which induces hepcidin (HEPC) secretion [16]. However, there are still scientific doubts as to how HFE and TfR2 might regulate hepcidin expression [17]. Hepcidin, a 25-amino-acid peptide hormone [18], binds to ferroportin, resulting in its internalization and degradation, preventing tissue Fe overload [15]. Erythroferrone (ErFe), a peptide hormone produced by erythroblasts, suppresses hepcidin expression, leading to increased Fe availability from the diet [19]. Small amounts of non-transferrin-bound iron (NTBI) are transported from the bloodstream into cells by the ZRT/IRT-like protein 14 (ZIP14) [20]. Tissue Fe is stored in the form of ferritin (Ft), mainly in the hepatic parenchyma. Ferritin acts as a tissue buffer against Fe deficiency and overload. Small amounts of ferritin are present in the bloodstream and serve as a diagnostic marker of somatic Fe levels [15]. Lactoferrin (LTF), a nonheme protein, also binds Fe in the form of Fe(III), regulating Fe metabolism and preventing Fe overload [21]. However, there are still doubts that lactoferrin plays a role in Fe metabolism. While it may bind Fe, its effects are predominantly antimicrobial and anti-inflammatory [22]. It has recently been demonstrated that the synthesis of homocysteine (HCY), a broadly investigated risk factor for cardiovascular diseases, is catalyzed by Fe. It is thus hypothesized that a homocysteine serum level is a marker of non-protein-bound Fe (free Fe) [23].

Maintaining proper Fe balance ensuring full health to the organism is dependent not only on adequate Fe supply, but also broadly on intestinal Fe alterations and absorption [24]. As there is no regulated Fe excretory mechanism, proper Fe absorption from the diet is crucial for its homeostasis [25]. It has recently been documented that intestinal dietary Fe and gut microbiota interact, leading to significant changes in Fe balance [26,27,28]. The gut microbiota decreases the quantity of Fe-binding substances converting Fe-binding ellagic acid into urolithin A [29] and turn Fe ions into Fe(II), an ionic form absorbable in the intestine [1]. Rodents lacking intestinal microbiota show an Fe deficit in intestinal cells and decreased intestinal absorption and retention [27,30]. Furthermore, antibiotic treatment significantly abates gut Fe absorption in rats and rabbits [15]. On the other hand, dietary Fe deficiency leads to a translocation of gut bacteria [28], decreased intestinal microbiota heterogeneity [31], and significant intestinal microbiota dysbiosis, as shown by alterations such as elevated abundance of Veillonellaceae and Enterobacteriaceae and decreased representation of Coriobacteriaceae [32]. However, the sophisticated crosstalk between the host’s intestine and gut bacteria, which affects Fe homeostasis, has not yet been sufficiently investigated [15]. It is well proven that the main site of iron absorption in the gut is the proximal small intestine. However, limited studies have revealed that in the intestinal parts of rats that are abundant in microbiota, such as the colon, the presence of DMT1 on enterocytes is elevated, which can ameliorate Fe gut absorption [33]. Lactoferrin, an Fe-binding glycoprotein, is able to decrease adhesion and invasion of *Shigella*, responsible for colon disorders [34]. Moreover, it has been documented that the intestinal microbiota regulates the gut expression of DCYTB, DMT1, TfR, and ferritin genes in epigenetic mode [15]. However, studies on gut microbiota and Fe metabolism are still scant. As gut microbiota is responsible for bioconversion of nutrients, Fe in this range, it is supposed that the loss of proper microbiota function may lead to disordered metabolism of microelements such as Fe [35]. Recently, it has been hypothesized that disturbed microbiota takes part in the development of a number of diseases, such as celiac disease [36] or gastric disorders [37].

In recent years, many studies have looked at how to ameliorate gut microbiota, with probiotics becoming the most effective intervention [38]. The World Health Organization (WHO) and the Food and Agriculture Organization (FAO) have defined probiotics as living microorganisms that beneficially affect the host’s health [39]. Recently, the favorable effect of probiotic supply on Fe homeostasis, including intestinal and liver Fe balance, has been shown [27,40]. In particular, *Lactobacillus plantarum* 299v (Lp299v) has been shown to exert beneficial influence on Fe metabolism. Bering et al. have documented that a lactic fermented (*Lactobacillus plantarum* 299v) oat gruel increased Fe absorption in healthy young females [41]. In addition, Hoppe et al. in a series of human studies documented improved Fe absorption as an effect of *Lactobacillus plantarum* 299v [42,43]. These and other studies became a background for health claim application to the European Food Safety Authority (EFSA) on increased Fe absorption as an effect of *Lactobacillus plantarum* 299v administration. However, EFSA did not approve the application stating that there is no sufficient evidence for the claim and that no plausible mechanism of *Lactobacillus plantarum* 299v was pointed [44]. EFSA refusal initiated scientific teams to perform further studies, not only human, but also in vitro [45] and animal [46], showing a beneficial effect of *Lactobacillus plantarum* 299v on Fe absorption. In our study, we have implemented multistrain probiotic consisted of nine bacterial strains: *Bifidobacterium bifidum* W23, *B. lactis* W51, *B. lactis* W52, *Lactobacillus acidophilus* W37, *L. brevis* W63, *L. casei* W56, *L. salivarius* W24, *Lactococcus lactis* W19, and *Lc. lactis* W58, in equal proportions. As the influence of *Lactobacillus plantarum* 299v on Fe metabolism is well investigated, in our study, we used a probiotic mixture with no addition of *Lactobacillus plantarum* 299v.

The WHO’s and FAO’s definitions state that the healthful benefits of probiotics are related to the choice of dose and need to be demonstrated for each strain separately [39]; as a result, the vast majority of studies on probiotics have employed one, or occasionally two, strains in a single dose.

In our previous paper [47] on multistrain probiotic supplementation in two doses in rats, we reported that, at the completion of supplementation, the liver mass of the rats was significantly smaller in groups receiving probiotics compared to the control group not receiving probiotics. In addition, the group receiving the higher dose of probiotics presented a significantly lower serum concentration of triglycerides and alanine transaminase (ALT), an important marker of liver function [48], compared to the control group. These results allowed us to state that multistrain probiotic supplementation does not disturb liver function and even exerts a favorable and dose-dependent effect on the liver. In our second paper, presenting further results of the same experiment [49], we showed that serum Fe was lower in both groups receiving probiotics compared to the control group. Moreover, Fe content in the liver was higher in the group supplemented with the higher dose of probiotics compared to control, and in the duodenum Fe content was higher in both supplemented groups compared to the control group. We concluded that oral multispecies probiotic supplementation induces an Fe shift from serum and intensifies liver Fe uptake. Intensified liver Fe uptake may lead to hepatic Fe overload [50]. It was shown that liver iron overload was associated with elevated blood levels of ALT [51]. However, in our previous studies, we did not find this relationship [47,49].

Based on our previous results [47,49], which showed the influence of multistrain probiotics on iron status in rats, in this study, we decided to explore the issue investigating biochemical markers of Fe balance. Thus, in the present paper, we analyzed the content of DMT1, TfR1, TfR2, and ZIP14 in liver homogenate samples and serum concentration of hepcidin, homocysteine, lactoferrin, ferritin, and erythroferrone. The present paper shows the results of implementation of the same probiotic mixture consisting of nine bacterial strains: *Bifidobacterium bifidum* W23, *B. lactis* W51, *B. lactis* W52, *Lactobacillus acidophilus* W37, *L. brevis* W63, *L. casei* W56, *L. salivarius* W24, *Lactococcus lactis* W19, and *Lc. lactis* W58, in equal proportions, which was presented in our former papers [47,49]. As shown in our previous publication [47,49], daily doses of supplemented probiotics were 2.5 × 10^9^ colony forming units (CFU) in the PA group (*n* = 10 rats) and 1 × 10^10^ CFU in the PB group (*n* = 10 rats) compared to placebo (KK group; *n* = 10 rats). The aim of our present study was to investigate the influence of six weeks of multispecies probiotic supply per os in two doses on selected Fe metabolism parameters in rats.

## 2. Materials and Methods

### 2.1. Animals

The study protocol was approved by the local animal studies bioethics committee (approval no. 24/2017) and conformed to protocols of Poznań University of Life Sciences, the Polish law on animal studies, the National Institutes of Health *Guide for the Care and Use of Laboratory Animals* (National Institutes of Health Publication No. 80–23, Revised 1978), and the European Communities Council Directive of 24 November 1986. Thirty male Wistar rats of the same strain, at the age of 10 weeks, were obtained from the Department of Toxicology at Poznań Medical University, Poland, directly before the experiment. This was the same set of rats we described in our previous papers [47,49]. The rats’ mean body mass at baseline was 263 ± 22 g [47]. Details of animal breeding conditions are presented in our previous papers [47,49].

### 2.2. Experimental Design

Using a random number generator, the study animals were randomized into 3 groups with 10 rats per group: KK, PA, and PB. The trial lasted 6 weeks. Throughout the experiment, the rats received a standard AIN-93M maintenance diet (Altromin, Lage, Germany). The Fe content in the diet was determined after digestion in 65% (*w*/*w*) spectra pure HNO_3_ (Merck, Kenilworth, NJ, USA) in a microwave digestion system (Speedwave Xpert, Berghof, Eningen, Germany). After digestion and dilution with deionized water, the Fe content in the mineral solution was determined using flame atomic absorption spectrometry (AAS-3, Carl Zeiss, Jena, Germany). The Fe content in the diet was measured at a wavelength of 248.3 nm. The PA and PB groups additionally received a multispecies probiotic in their diet at a dose of 2.5 × 10^9^ CFU/day (PA) or 1 × 10^10^ CFU/day (PB). No probiotic was added to the diet of the control group (KK). Further details of the experimental design are presented in our previous papers [47,49].

### 2.3. Probiotic

A probiotic mixture of 9 bacterial strains at a dose of 2.5 × 10^9^ CFU/g (*Bifidobacterium bifidum* W23, *B. lactis* W51, *B. lactis* W52, *Lactobacillus acidophilus* W37, *L. brevis* W63, *L. casei* W56, *L. salivarius* W24, *Lactococcus lactis* W19, and *Lc. lactis* W58, in equal proportions) was dispersed directly into a ration of the diet. The specific probiotic mixture was Ecologic Barrier (Winclove Probiotics, Amsterdam, Netherlands) [52]. Probiotics administered to rats were active. This could be seen by differences in total fecal bacteria content between supplemented groups (higher in PB group) and increasing (although insignificantly) Lactobacillus fecal content with increased probiotic dose [49]. Details of supplemented probiotics are presented in our previous papers [47,49].

### 2.4. Blood and Liver Collection

Euthanasia of rats by carbon dioxide inhalation, preceded by body mass measurement, took place after 6 weeks of the trial. All animals were euthanized during the same time period each day, in the morning. Blood samples were collected in serum-separated tubes by cardiac puncture after 12 h fasting in order to obtain serum. During sectioning, the liver was removed, washed in saline, weighed, and stored at −20 °C. Whole blood was also collected. Detailed procedures of blood and liver sample collection are described in our previous papers [47,49].

### 2.5. Biochemical and Mineral Measurements

The concentrations of HEPC, LTF, HCY, Ft, and ErFe in the collected serum were determined using enzyme-linked immunosorbent assay (ELISA). Commercial ELISA kits were employed (Fine Test, Wuhan Fine Biological Technology, Hubei, China) and absorption spectrophotometry was used (LEDetect96, Labexim, Lengau, Austria). The accuracy of the concentration measurements was checked in each case by the following procedure: 2 standards were run as samples, and their calculated concentrations were compared to nominal ones; additionally, 3 randomly chosen rat samples were run in triplicate and their concentrations were compared. In each procedure, coefficient of variance did not exceed 5%. Reproducibility was verified using the control serum sample provided by the kit producer. Serum C-reactive protein (CRP) was measured at a commercial laboratory.

Liver sample homogenates were prepared using an automatic homogenizer (MagNALyser, Roche, Basel, Switzerland). The concentrations of DMT1, TfR1, TfR2, and ZIP14 in the liver homogenate samples were estimated using commercial ELISA kits (Shanghai Qayee Biotechnology, Shanghai, China, for DMT1, TfR1, and TfR2; Bioassay Technology Laboratory, Shanghai, China, for ZIP14).

### 2.6. Statistical Analysis

The data are shown as arithmetic means ± standard deviations. The Shapiro–Wilk test was used to check if the variables were distributed normally. The groups were compared using ANOVA with Tukey’s post hoc test. A Pearson correlation test was carried out to calculate the correlation coefficients. A *p*-value of less than 0.05 was regarded as significant. Statistica for Windows 10.0 was used (StatSoft, Kraków, Poland). It was calculated that a sample size of 10 rats in each group would yield 80% power of detecting statistical significance at the 0.05 α level.

## 3. Results

Serum concentrations of the Fe metabolism parameters (hepcidin, lactoferrin, homocysteine, ferritin, and erythroferrone) are presented in Table 1. No significant differences were found in hepcidin, lactoferrin, homocysteine, ferritin, and erythroferrone serum concentrations between the three groups. Thus, we saw no effect of the probiotic dose on these parameters. In all study rats, serum CRP level was below 1.0 mg/L, indicating no inflammatory state [53]. The levels of DMT1, TfR1, TfR2, and ZIP14 proteins in the liver are presented in Table 2. There were no significant differences between the three groups in terms of the content of DMT1, TfR1, TfR2, or ZIP14 protein in the liver. However, in our previous paper [49], we found a range of significant differences in Fe distribution following probiotic supplementation: serum Fe was lower in both PA and PB groups vs. KK group, and Fe content in the liver was higher in the PB group vs. the KK group [49]. Fe content in the diet was 56 mg/kg.

**Figure 1:**
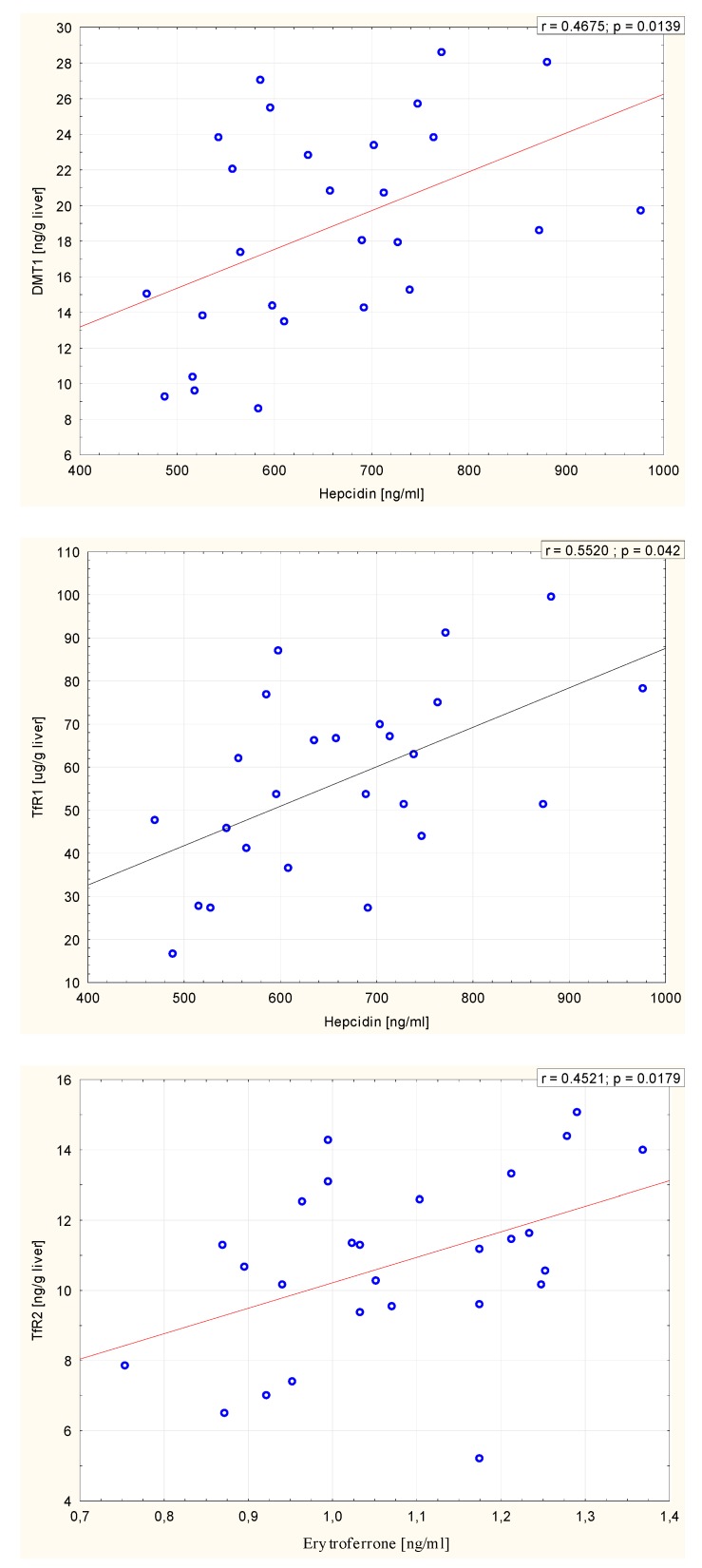
Significant correlations between serum hepcidin and hepatic divalent metal transporter 1 (DMT1), serum hepcidin and hepatic transferrin receptor 1 (TfR1), and serum erythroferrone and TfR2.

Correlation analysis of the overall sample (*n* = 30 rats) revealed a range of positive correlations between the serum concentrations of hepcidin and lactoferrin, and hepcidin and ferritin. Interestingly, in the overall sample (*n* = 30), a positive correlation was found between the serum concentrations of hepcidin and DMT1 in the liver, hepcidin, and TfR1 in the liver, and erythroferrone and TfR2 in the liver. Moreover, positive correlations were seen in all rats (*n* = 30) between the hepatic content of DMT1 and TfR1, and TfR1 and TfR2, and a negative correlation between DMT1 and ZIP14. The significant correlations found in the overall sample (*n* = 30 rats) are presented in Table 3 and Figure 1.

## 4. Discussion

Despite the research to date, still not much is known about the mechanisms by which probiotic supplementation affects Fe homeostasis and the lack of the knowledge in this range has been pointed by EFSA [44]. However, some pathways for which probiotics impact iron metabolism have been investigated. In an in vitro study, Sandberg et al. has shown that a probiotic *Lactobacillus plantarum* 299v (Lp299v) effect on Fe absorption is mediated through the Fe^3+^/DCYTB axis–ferric reductase DCYTB is increased in the presence of *Lactobacillus plantarum* 299v (Lp299v) [45]. In an animal study, Adiki et al. documented that probiotic *Lactobacillus plantarum* 299v (Lp299v) increases Fe absorption from the diet, and this effect was independent from probiotic dose [46]. In human studies, Hoppe et al. [43] have shown that intake of *Lactobacillus plantarum* 299v (Lp299v) ameliorates iron absorption from a fruit drink. Authors hypothesize that this effect may be related to the colonization of the bacteria in the gut or increased mucin excretion caused by *Lactobacillus plantarum* 299v (Lp299v) [54]. Another possible mechanism of increased Fe absorption caused by probiotics can be decrease in intestinal pH, leading to the reduction of ferric Fe into absorbable ferrous Fe as an effect of lactobacilli growth [55]. However, other studies did not confirm this mechanism [56,57]. In addition, Hoppe et al. documented that freeze-dried *Lactobacillus plantarum* 299v (Lp299v) increases the absorption of Fe when administered with a meal [42]. Recently, it has been postulated that probiotic Lactobacillus, due to synthesized p-hydroxyphenyllactic acid, shows a ferric-reducing activity, which also intensifies duodenal Fe absorption [26,58]. In our study, we used multistrain probiotic consisting of nine bacterial strains: *Bifidobacterium bifidum* W23, *B. lactis* W51, *B. lactis* W52, *Lactobacillus acidophilus* W37, *L. brevis* W63, *L. casei* W56, *L. salivarius* W24, *Lactococcus lactis* W19, and *Lc. lactis* W58, in equal proportions. As the influence of *Lactobacillus plantarum* 299v on Fe metabolism is well investigated [41,42,43], in our study, we used probiotic mixture with no addition of *Lactobacillus plantarum* 299v (Lp299v).

In our previous paper, we reported higher hepatic and duodenal Fe levels and lower serum Fe levels as effects of probiotic supply [49]. In this study, we attempted to investigate the effect of probiotic supply on Fe balance using a more analytical approach. The novelty of our study is the use of dose-comparison mode and multistrain probiotics, which is unique among studies of Fe metabolism. Moreover, we investigated the effects of probiotic supply on erythroferrone, a scarcely investigated marker of Fe balance. In addition, we show a range of significant correlations between biochemical factors involved in Fe turnover, which have not been previously reported in conditions of multistrain probiotic supplementation. The findings presented in this paper significantly increase our knowledge of Fe balance in conditions of probiotic supply and allow us to state that probiotic supplementation with probiotic mixture with no addition of *Lactobacillus plantarum* 299v (Lp299v) does not disturb investigated biochemical regulatory mechanisms responsible for Fe metabolism, despite a previously reported increase in liver Fe stores, which we have shown in experimental study [49].

A multistrain probiotic supplement administered in the study to rats was active. This could be seen by total fecal bacteria content higher in PB group compared to PA group and increasing (although insignificantly) Lactobacillus fecal content in feces with increased probiotic dose [49]. Thus, it should be stated that probiotic supplements used in the study fulfilled the viability condition given in the WHO probiotic definition [39]. Our previous study [49] revealed a significant effect of multistrain probiotic supplementation on Fe metabolism. This former study [49] revealed a favorable effect of oral multispecies probiotic supplementation on Fe availability and duodenal Fe absorption. Moreover, multistrain probiotic resulted in Fe shift from serum and intensified pancreatic and liver iron uptake. These outcomes, accompanied by differences in total fecal bacteria content and Lactobacillus fecal content (although insignificant) between groups [49], have shown that, in relation to a timing of gut colonization by probiotic bacteria, a period of 6 weeks was long enough to induce significant iron-related response. Hoppe et al. [43] have documented significant changes in Fe-metabolism in response to probiotic supplementation after a period as short as four days.

The most relevant result of our study presented in this paper is the lack of influence of probiotic supplementation on Fe metabolism in the range of analyzed parameters. Neither the daily dose of 2.5 × 10^9^ CFU nor 1 × 10^10^ CFU exerted the significant influence on serum concentration of ferritin, hepcidin, erythroferrone, lactoferrin, and homocystein. As serum ferritin is the most important marker of body Fe status [15], it should be stated that total Fe body stores in response to probiotic supply remained unchanged. Furthermore, the experiment revealed no differences between probiotic and placebo supplemented groups in the range of liver Fe-related molecules: DMT1, TfR1, TfR2, ZIP14, independently from probiotic dose. This shows that probiotic supply with probiotic mixture with no addition of *Lactobacillus plantarum* 299v (Lp299v) implemented in the study did not affect Fe homeostasis.

Somatic Fe is stored mainly in the liver parenchyma in the form of ferritin [15]. In this study, we found no differences in serum ferritin concentration between study and control groups. We did, however, show that multistrain probiotic supplementation in the PA group resulted in insignificantly higher liver levels of DMT1 and TfR1 than in the PB and KK groups (DMT1: *p* = 0.7410 for PA vs. PB and *p* = 0.7391 for PA vs. KK; TfR1: *p* = 0.9558 for PA vs. PB and *p* = 0.9920 for PA vs. KK). Nam et al. [20] reported that DMT1 content in the liver was 200% higher in Fe-deficient rats and 70% lower in Fe-loaded rats than in Fe-adequate rats. They similarly noted that the hepatic level of TfR1 increased in Fe-deficient animals and decreased in Fe-loaded rats. On the other hand, hepatic ZIP14 content increased in Fe-loaded and hepatic Fe-overloaded rats as compared to Fe-adequate ones [20]. In the present study, we observed that that hepatic content of ZIP14 did not differ between study groups: in the PB group, it was even insignificantly lower than in the KK and PA groups (*p* = 0.9869 for PB vs. KK and *p* = 0.9789 for PB vs. PA). Our results thus demonstrate that multistrain probiotic supplementation did not lead to hepatic Fe overload.

We show here that the hepatic content of TfR2 was higher in the PA group than in the PB and control groups, though the difference did not reach the level of significance. After binding HFE translocated from TfR1, TfR2 induces hepcidin synthesis, which should lead to increased hepcidin serum concentration [16]. However, we did not observe altered hepcidin serum content, nor did we observe significant differences in the erythroferrone serum level between the three groups. Studies on β-thalassemia have revealed that erythroferrone contributes to Fe overload in conditions of ineffective erythropoiesis [59,60]. It also acts as an hepcidin suppressor during stress erythropoiesis [60]. Based on our results, we can state that multistrain probiotic supplementation does not disturb the interaction between hepcidin and erythroferrone, does not disrupt Fe homeostasis, and does not lead to Fe overload, despite increased hepatic Fe content [15]. This demonstrates the safety of probiotic supplementation in the range of Fe balance.

Interestingly, we noted a range of significant correlations in the overall sample (*n* = 30) that have not been reported previously in any trial with probiotic intervention. The serum hepcidin level correlated positively with hepatic DMT1 and TfR1 content, and there was also a positive correlation between hepatic levels of DMT1 and TfR1, accompanied by a negative correlation between the hepatic levels of DMT1 and ZIP14. The positive correlations of hepcidin with DMT1, of hepcidin with TfR1, and of DMT1 with TfR1 represent a physiological mechanism of well-balanced Fe homeostasis and prevent an excess of Fe in the blood [12,15,20]. Furthermore, we show here positive correlation between serum ferritin and hepcidin concentrations—mechanisms preventing serum and tissue Fe overload [15], which hypothetically led to the previously noted increased duodenal Fe accumulation [49]. Our result confirms the undisturbed hepcidin action and proper functioning of mechanisms that prevented Fe excess in our study population.

We noted a positive correlation between serum hepcidin and lactoferrin, a globular glycoprotein with strong Fe-binding and antimicrobial properties [61,62]. It has been demonstrated that lactoferrin does not present antibacterial activity against probiotics [63]. The lack of difference in lactoferrin blood concentration between the study and control groups found in our trial shows that the administration of probiotics has no effect on lactoferrin levels.

However, it should be stated that, despite *p*-values of presented correlations indicating their significance, the r-values are quite low. This shows that associations between analyzed molecules are not strong and the issue of interdependencies found in the study requires further investigation.

In our previous study [49], we found that multistrain probiotic supplementation led to increased duodenal and liver Fe content and decreased serum Fe concentration. The results of that study seem to be coherent with the results presented in this paper. Despite decreased serum Fe content and increased liver Fe level, in this study, we did not find differences in ferritin serum level between groups. This suggests that what we observed was an Fe shift—Fe present in serum has been translocated to the liver, and total Fe body stores remained stable. Interestingly, previously, we registered an increased Fe duodenal level as a result of probiotic supply. We hypothesize that this was an effect of increased diet Fe absorption with stable, or even decreased, Fe transport from duodenal cells to the bloodstream. However, this should be accompanied by altered hepcidin and erythroferrone serum levels, which was not observed in the study. Undoubtedly, such results need further investigation and the lack of significant differences between groups in the range of selected biochemical parameters presented in this paper might be an effect of a too-short supplementation period, as we stated in the section on limitations of the study (below). On the other hand, the lack of difference in serum hepcidin and erythroferrone levels between groups confirms our hypothesis that, despite higher Fe content in the liver in the PB group compared to control, multistrain probiotic supplementation did not lead to Fe overload. Furthermore, Fe overload is correlated not only with intensified hepcidin production, but also with inflammation. In our trial, we observed low levels of serum CRP in all groups of rats (i.e., below 1.0 mg/L), which indicates no inflammatory state in the study animals and seems to further confirm our hypothesis of no hepatic Fe excess and undisturbed biochemical regulatory mechanisms responsible for Fe balance in our study [15,49].

Non-protein-bound Fe is a catalyst for oxygen free radical synthesis, a causative factor of intensified oxidative stress with great detrimental health consequences. Recently, homocysteine serum concentration has been identified not only as an indicator of a non-protein-bound Fe level, but also as a marker of oxidative stress and cardiovascular risk [23]. Our study found no differences in homocysteine serum content between the three groups of rats. Thus, we can presume that multistrain probiotic supplementation does not affect non-protein-bound Fe serum content.

### 4.1. Strong Points of the Study

The strongest point of our study is its analytical approach, which aims to clarify the biochemical basis of the effect of probiotic supplementation on Fe metabolism [49]. Moreover, we administered the probiotic supplementation in two doses in rats without a previously induced Fe deficit and with no Fe supply, which had not been done in any previous trial. In addition, the animal study approach allowed us to determine the levels of Fe metabolism-related molecules such as DMT1, ZIP14, TfR1, and TfR2 in the hepatic tissue. No such analysis had previously been carried out under conditions of probiotic supply. Finally, our trial succeeded in showing significant correlations of the hepatic concentrations of these molecules. Our study shows undisturbed Fe regulatory mechanisms in conditions of multistrain probiotic supply.

### 4.2. Limitations of the Study 

One significant limitation of our study is that we did not analyze a range of molecules that take part in Fe turnover, such as HFE, ferroportin, and NTBI. This suggests the possibility of further studies in the field of probiotic supply and Fe homeostasis. The study period of six weeks was also perhaps not sufficient to reveal a range of significant results. In particular, we did not analyze feces for Fe content. This should be done in future trials. Some studies have demonstrated that hepcidin levels can be affected by prolonged fasting [64,65]. Thus, using a fasting period in our trial may constitute a limitation in reference to determining hepcidin serum levels. In addition, we have not analyzed the content of Fe metabolism related molecules in the intestine, duodenum in this range. However, in the experiment samples, duodenum has been used to determine the Fe content, which has been presented in our previous paper [49]. This points to the directions of future study in the range of probiotics and Fe homeostasis. Moreover, multistrain probiotic supplement implemented in the study did not include *Lactobacillus plantarum* 299v (Lp299v), which has been shown to impact iron absorption in both humans and animals.

## 5. Conclusions

Six weeks of oral supplementation with multistrain probiotic consisting of nine bacterial strains: *Bifidobacterium bifidum* W23, *B. lactis* W51, *B. lactis* W52, *Lactobacillus acidophilus* W37, *L. brevis* W63, *L. casei* W56, *L. salivarius* W24, *Lactococcus lactis* W19, and *Lc. lactis* W58, in equal proportions in two doses (2.5 × 10^9^ CFU/day, *n* = 10 rats and 1 × 10^10^ CFU/day, *n* = 10 rats) did not demonstrate a significant influence on selected parameters of Fe metabolism. The lack of significant effect of the particular multistrain probiotic used in the study on selected parameters of Fe metabolism could have resulted from no *Lactobacillus plantarum* 299v (Lp299v) addition to the probiotic mixture. The positive correlations of serum hepcidin and liver divalent metal transporter 1, serum hepcidin and liver transferrin receptor 1, and serum erythroferrone and liver transferrin receptor 2 seem to represent a range of significant interdependencies in the field of somatic Fe homeostasis. Further studies on a larger scale are needed to draw more precise conclusions.

## Figures and Tables

**Table 1:** Serum Fe metabolism parameters.

Group	n	Hepcidin (ng/mL)	*p*-Value	Lactoferrin (pg/mL)	*p*-Value	Homocystein (pmol/mL)	*p*-Value	Ferritin (pg/mL)	*p*-Value	Erythroferrone (ng/mL)	*p*-Value
**KK**	10	692.25 ± 144.99	KK vs. PA 0.8518	107.47 ± 25.27	KK vs. PA 0.5660	39.46 ± 11.57	KK vs. PA 0.7570	64.17 ± 3.70	KK vs. PA 0.9172	0.98 ± 0.19	KK vs. PA 0.3596
**PA**	10	659.60 ± 124.36	KK vs. PB 0.4693	97.42 ± 23.41	KK vs. PB 0.1670	35.99 ± 8.10	KK vs. PB 0.2384	64.96 ± 4.95	KK vs. PB 0.8428	1.08 ± 0.12	KK vs. PB 0.1005
**PB**	10	620.40 ± 114.41	PA vs. PB 0.7719	89.16 ± 11.52	PA vs. PB 0.6472	31.38 ± 11.03	PA vs. PB 0.5793	63.06 ± 3.69	PA vs. PB 0.5751	1.14 ± 0.13	PA vs. PB 0.7061

Data are presented as mean ± standard deviation (SD). KK, control group; PA, group with low dose of probiotic; PB, group with high dose of probiotic. One-way ANOVA with Tukey’s post hoc test was performed.

**Table 2:** Fe transporter proteins in the liver.

Group	n	DMT1 (ng/g)	*p*-Value	TfR1 (µg/g)	*p*-Value	TfR2 (ng/g)	*p*-Value	ZIP14 (ng/g)	*p*-Value
**KK**	10	17.87 ± 5.60	KK vs. PA 0.7391	56.95 ± 22.91	KK vs. PA 0.9920	10.98 ± 2.86	KK vs. PA 0.9628	14.83 ± 4.93	KK vs. PA 0.9997
**PA**	10	20.11 ± 5.78	KK vs. PB 0.9972	58.28 ± 24.27	KK vs. PB 0.9881	11.32 ± 2.83	KK vs. PB 0.9530	14.90 ± 4.88	KK vs. PB 0.9869
**PB**	10	18.08 ± 6.69	PA vs. PB 0.7410	55.25 ± 18.66	PA vs. PB 0.9558	10.60 ± 2.76	PA vs. PB 0.8254	14.40 ± 5.62	PA vs. PB 0.9789

Data are presented as mean ± SD. DMT, divalent metal transporter 1; KK, control group; PA, group with low dose of probiotic; PB, group with high dose of probiotic; TfR1, transferrin receptor protein 1; TfR2, transferrin receptor protein 2; ZIP14, ZRT/IRT-like protein 14. One-way ANOVA with Tukey’s post hoc test was performed.

**Table 3:** **Significant** (*p* < 0.05) correlations registered in the study.

Correlated Parameters	r	*p*-Value
HEPC–LTF	0.50	0.01
HEPC–Ft	0.47	0.023
DMT1–TfR 1	0.69	<0.001
DMT1–ZIP14	−0.50	0.044
TfR1–TfR 2	0.69	<0.001

DMT1, divalent metal transporter 1; ErFe, erytroferrone; Ft, ferritin; HEPC, hepcidin; LTF, lactoferrin; TfR1, transferrin receptor protein 1; TfR2, transferrin receptor protein 2; ZIP14, ZRT/IRT-like protein 14. Pearson correlation test was performed.

## Research Involving Animals

All applicable international, national, and institutional guidelines for the care and use of animals were followed. All procedures performed in the studies involving animals were in accordance with the ethical standards of the institution or practice at which the studies were conducted. All of the experimental procedures were approved by the local bioethics committee for animal studies (approval No. 24/2017).

## Abbreviations

ALT	alanine transaminase
CRP	C-reactive protein
CV	cardiovascular
DCYTB	membrane-bound ferrireductases
DMT1	divalent metal transporter
EFSA	European Food Safety Authority
ELISA	enzyme-linked immunosorbent assay
ErFe	erythroferrone
FAO	Food and Agriculture Organization
Ft	ferritin
Fe	iron
free Fe	non-protein-bound Fe
HCY	homocysteine
HEPC	hepcidin
HFE	TfR1-associated protein
Hgb	hemoglobin
LTF	lactoferrin
NTBI	non-transferrin-bound iron
TfR1	transferrin receptor 1
TfR2	transferrin receptor 2
WHO	World Health Organization
ZIP14	ZRT/IRT-like protein 14
